# Erythema Multiforme Associated With Pembrolizumab Treatment for Recurrent Urothelial Carcinoma: A Case Report

**DOI:** 10.7759/cureus.105345

**Published:** 2026-03-16

**Authors:** Carolina Ledesma-Félix, Sofía Valdés-Loperena, Sofía E Márquez-Macedo, Michelle Gatica-Torres, Sergio E Leal-Osuna

**Affiliations:** 1 Internal Medicine, Hospital Español de México, Mexico City, MEX; 2 Clinical Sciences, Tecnológico de Monterrey, Mexico City, MEX; 3 Dermatology, Hospital Español de México, Mexico City, MEX

**Keywords:** bladder cancer, erythema multiforme, immune checkpoint inhibitors, immune-related adverse events, pembrolizumab

## Abstract

Immune checkpoint inhibitors (ICIs) have significantly improved outcomes in patients with advanced malignancies; however, their use is associated with immune-related adverse events (irAEs) that may affect multiple organ systems. Cutaneous manifestations are the most common and often represent the earliest signs of immune dysregulation. Erythema multiforme (EM) is a rare, but potentially underrecognized, cutaneous adverse event associated with ICI therapy.

We report the case of an 84-year-old woman with recurrent urothelial carcinoma who developed EM following the sixth cycle of pembrolizumab. The eruption presented as symmetrically distributed, annular erythematous plaques with central crusting on the upper extremities and trunk, without mucosal involvement or systemic symptoms. Histopathologic examination revealed vacuolar interface dermatitis with lymphocytic infiltration, supporting the diagnosis. The patient was successfully managed with systemic and topical corticosteroids, along with supportive skin care, resulting in complete resolution of lesions. Pembrolizumab was subsequently discontinued, and no recurrence of cutaneous findings was observed.

This case highlights the importance of recognizing EM as a rare, immune-related cutaneous adverse event associated with pembrolizumab. Early identification and appropriate management are essential to prevent progression and minimize interruptions in oncologic therapy. Increased awareness among clinicians may facilitate timely diagnosis and improve patient outcomes as the use of immunotherapy continues to expand.

## Introduction

In recent years, the development of immunotherapeutic drugs targeting immune checkpoint pathways has revolutionized cancer therapy, providing durable responses in malignancies that were previously considered refractory to treatment. Programmed cell death protein 1 (PD-1) and its ligand (PD-L1) are key inhibitory molecules that maintain immune homeostasis by downregulating T-cell activity and preventing excessive immune responses. Some tumors express these molecules in order to evade the host immune system. The blockade of these pathways, through monoclonal antibodies such as pembrolizumab and nivolumab, activates T-cell cytotoxic function, leading to antitumor effects. Nevertheless, this same mechanism can break immune tolerance and result in immune-related adverse events (irAEs) [1].

Among the various organ systems affected by irAEs, the skin is the most commonly involved and of the earliest onset [1,2]. Reported dermatologic adverse events include maculopapular rash, pruritus, vitiligo, lichenoid dermatitis, psoriasis exacerbation, and pustulosis [2,3].

Erythema multiforme (EM) is an acute, immune-mediated mucocutaneous reaction characterized by the abrupt onset of targetoid erythematous lesions, typically distributed on the extremities, and sometimes associated with mucosal involvement. Although most cases are triggered by infections or medications, EM has also been described as a rare adverse event associated with immune checkpoint inhibitors (ICIs) such as ipilimumab, nivolumab, and pembrolizumab [4]. Clinically, distinguishing EM from Stevens-Johnson syndrome (SJS) and toxic epidermal necrolysis (TEN) is essential, as these conditions differ significantly in severity, prognosis, and management.

We describe the case of a patient who developed EM following pembrolizumab therapy for recurrent urothelial carcinoma, and provide a literature review on this uncommon adverse effect. Understanding and acknowledging different irAEs is essential for dermatologists and oncologists to ensure early recognition, appropriate management, and the safe continuation of immunotherapy when feasible.

## Case presentation

An 84-year-old woman with a past medical history of hypertension and dyslipidemia, treated with amlodipine and rosuvastatin, and breast cancer, diagnosed 17 years earlier and currently in remission, presented for evaluation. She also had a history of urothelial bladder carcinoma, initially diagnosed 14 years earlier, for which she underwent transurethral resection of the bladder tumor (TURBT) at the time of diagnosis. Six months prior to presentation, recurrence was confirmed by biopsy, demonstrating high-grade invasive urothelial carcinoma with squamous metaplasia. The patient subsequently completed six cycles of pembrolizumab, administered at three-week intervals, representing her first exposure to an ICI.

**Figure 1:**
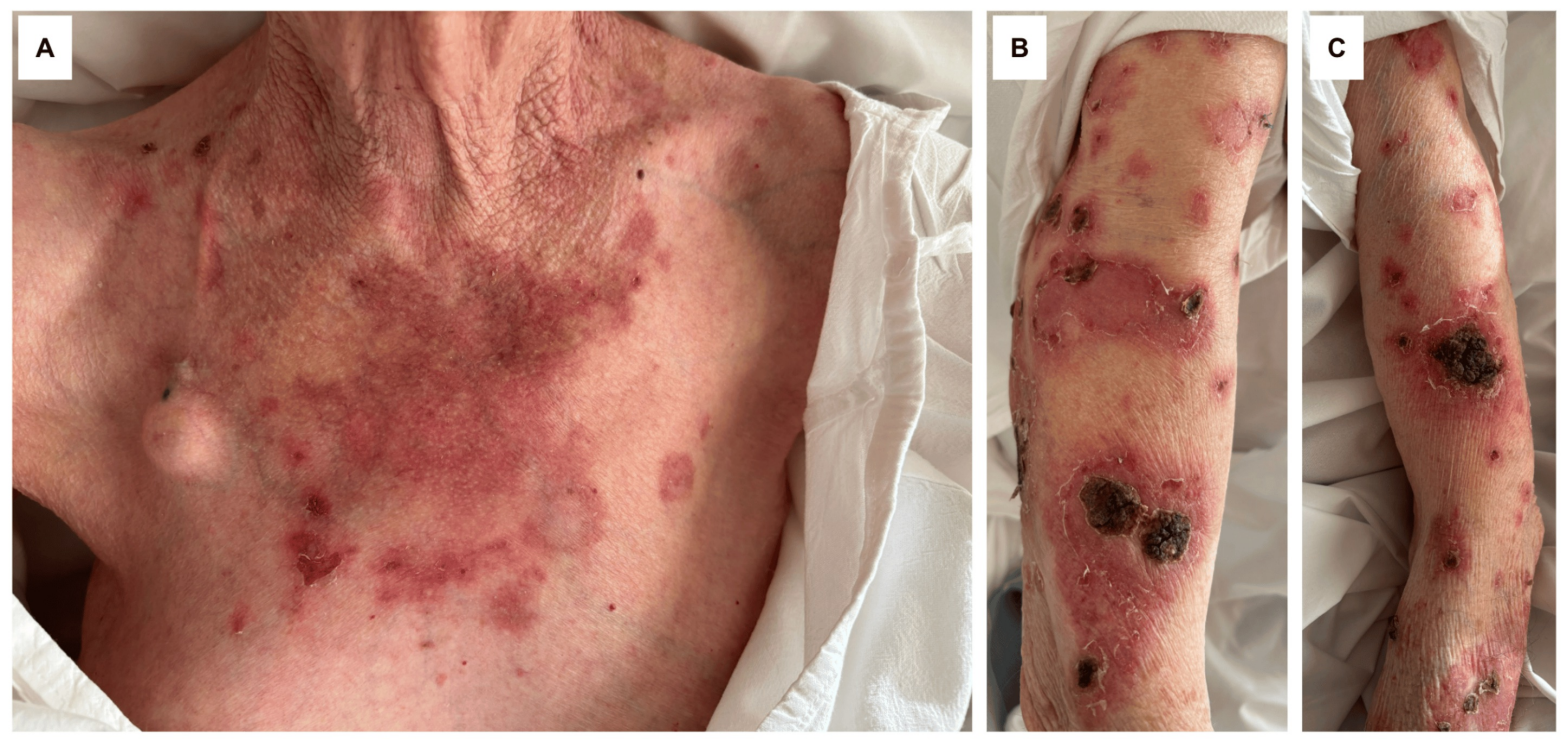
Clinical presentation of the cutaneous eruption A) Multiple annular erythematous plaques, with central crusting, involving the anterior chest. B) Similar lesions on the right upper extremity, showing well-demarcated erythematous plaques, with partial confluence. C) Symmetric involvement of the left upper extremity, with annular plaques and areas of central crusting.

Approximately 48 hours after the sixth 200 mg infusion of pembrolizumab, she developed a disseminated, bilaterally symmetrical eruption affecting the upper extremities and anterior trunk. The lesions consisted of multiple annular erythematous plaques, measuring 5-10 cm in diameter, some confluent, with central crusting or ulceration (Figure 1). No mucosal involvement or systemic symptoms, such as fever or malaise, were present.

**Figure 2:**
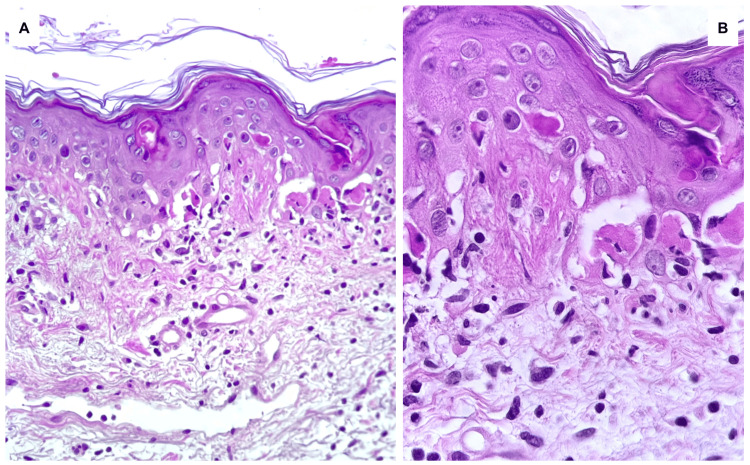
Histopathologic findings A) Hematoxylin and eosin stain (H&E, 100×), showing scattered necrotic keratinocytes throughout the epidermis, and vacuolar degeneration of the basal layer. B) Higher magnification (H&E, 400×), demonstrating confluent necrotic keratinocytes and a superficial perivascular inflammatory infiltrate, composed predominantly of lymphocytes and histiocytes, consistent with an interface dermatitis pattern compatible with erythema multiforme.

Initial management included 50 mg of oral prednisone daily. Dermatologic evaluation and an incisional skin biopsy from the right arm were performed. Histopathology revealed vacuolar interface dermatitis with lymphocytic infiltration (Figure 2). Differential diagnoses included EM, SJS/TEN, and immune-mediated cutaneous toxicity. Correlation with the clinical morphology, topographic distribution, and absence of mucosal involvement supported the diagnosis of EM minor. Treatment was continued with topical clobetasol propionate 0.05%, colloid soaks, to facilitate crust removal, and emollients. Based on the clinical history, absence of suggestive infectious symptoms, and lack of exposure to other medications commonly associated with EM, alternative triggers were considered unlikely. The lesions resolved gradually, with no new eruptions. No additional cycles of pembrolizumab have been administered.

## Discussion

Pembrolizumab is a humanized IgG4κ monoclonal antibody that selectively targets PD-1, preventing its binding with PD-L1 and PD-L2, and interrupting inhibitory signaling in T cells. This blockade enhances T-cell activation and proliferation, increasing the immune system’s ability to recognize and destroy tumor cells. However, this loss of inhibitory signaling may also impair self-tolerance, leading to the emergence of inflammatory or autoimmune events [5].

Cutaneous irAEs are hypothesized to result from the activation of cytotoxic T-cells targeting non-tumor cells, along with the formation of autoantibodies and the release of proinflammatory cytokines. This mechanism is thought to be shared among irAEs affecting different organs. The incidence of irAEs does not depend on tumor type, but on the specific checkpoint inhibitor used. Organs whose immune homeostasis is maintained primarily by peripheral T-cell tolerance, including the skin and colon, are particularly prone to developing irAEs [1]. 

Dermatologic toxicities occur in up to 40% of patients receiving ICIs targeting the PD-1/PD-L1 pathway, and usually appear within the first two treatment cycles [6]. Specifically for pembrolizumab, dermatologic adverse events have been reported in approximately 14% of treated patients [2]. Interestingly, in some malignancies, such as melanoma, their occurrence - particularly vitiligo - has been associated with improved prognosis and longer overall survival, suggesting that a heightened immune response may reflect effective tumor recognition [7]. Sanlorenzo et al. reported similar findings in patients receiving pembrolizumab across different malignancies, including melanoma, lung cancer, prostate cancer, and Merkel cell carcinoma, regardless of the type of cutaneous irAE observed [3].

Most cases are mild and do not require treatment interruption, though rare, severe reactions, such as SJS/TEN, drug reaction with eosinophilia and systemic symptoms (DRESS), and EM, have been documented. In these cases, treatment may include systemic immunosuppressants and treatment interruption [7].

The estimated incidence of EM among ICI-treated patients is 0.1%. While drug-induced EM typically appears within three days of exposure, immunotherapy-related EM often shows a delayed onset. A large U.S. cohort described a median onset of 106 days after treatment initiation [8]. A systematic review found a mean time of onset of 28 days in patients receiving treatment with nivolumab, though no pembrolizumab-associated cases were reported [9].

Silvi et al. described a 77-year-old man with recurrent oral squamous cell carcinoma, who developed EM after the third pembrolizumab infusion, which resolved after oral prednisolone without requiring discontinuation of immunotherapy. Similarly, our patient presented with EM after multiple cycles, highlighting that delayed hypersensibility mechanisms may be involved [10].

Some authors suggest that the mechanism by which PD-1/PD-L1 inhibitors can cause EM is through upregulation of FAS receptors on keratinocytes, increasing their susceptibility to apoptosis through FAS-FAS ligand interactions, a pathway implicated in EM pathogenesis [4].

Histopathologic findings include colloid bodies at the dermoepidermal junction, suprabasal vacuolar degeneration, and a superficial perivascular infiltrate of lymphocytes, histiocytes, melanophages, and eosinophils, consistent with interface dermatitis [10]. In our case, biopsy revealed vacuolar interface dermatitis, a reactive pattern requiring clinical correlation.

Treatment strategies for pembrolizumab-induced EM have included oral prednisone, topical corticosteroids (triamcinolone 0.1%, bethamethasone 0.05%, clobetasol 0.05%), and, in severe cases, intravenous methylprednisolone pulses [10]. In the cohort by Wongvibulsin et al. [8], none of the cases of EM associated with ICIs received systemic immunosuppression within seven days of diagnosis. Current European Society for Medical Oncology (ESMO) and National Comprehensive Cancer Network (NCCN) guidelines for management of ICI-related toxicities do not provide EM-specific recommendations, but advise topical corticosteroids for mild cutaneous toxicities, systemic corticosteroids for moderate to severe cases, and permanent discontinuation of the offending agent in life-threatening reactions. Supportive measures, such as antihistamines, may provide symptomatic relief from pruritus and discomfort [6,11].

## Conclusions

As the clinical use of ICIs continues to expand, clinicians must remain vigilant for an increasingly broad spectrum of irAEs. EM associated with pembrolizumab is rare, but clinically relevant, and this case highlights that cutaneous immune-related reactions may occur after several treatment cycles, underscoring the need for continued monitoring throughout therapy. Importantly, accurate differentiation between EM and more severe reactions, such as SJS/TEN, is essential, as these entities differ significantly in prognosis and management.

This report emphasizes the key role of multidisciplinary collaboration between dermatologists and oncologists in the recognition and management of immune-related cutaneous toxicities. Early identification and appropriate immunosuppressive therapy can control symptoms, prevent progression, and help preserve the continuity of oncologic treatment when feasible. Finally, additional well-documented clinical reports are needed to better define the incidence, clinical spectrum, and optimal management of pembrolizumab-associated EM, thereby improving recognition and care of this uncommon but meaningful adverse event.
